# Effect of plant-soil system on the restoration of community stability after wildfire in the northeast margin of Qinghai-Tibet plateau

**DOI:** 10.1038/s41598-024-61621-2

**Published:** 2024-05-10

**Authors:** Zizhen Li, Jia Wei, Wanpeng He, Xueping Cao, Xiaolei Zhou, Qing Tian

**Affiliations:** 1 College of Forestry, Gansu Agricultural University, Lanzhou, China; 2 Gansu Academy of Agricultural Sciences, Lanzhou, China; 3 Research Institute of Forestry, Chinese Academy of Forestry, Beijing, China

**Keywords:** Forest ecosystems, Post-fire recovery, Plant-soil feedback, Partial least squares path modeling, Qinghai-Tibet Plateau, Ecology, Environmental sciences, Natural hazards

## Abstract

Wildfires, as an environmental filter, are pivotal ecological disturbances that reshape plant communities and soil dynamics, playing a crucial role in regulating biogeographic patterns and ecosystem services. In this study, we aim to explore the effects of wildfires on forest ecosystems, specifically focusing on the plant-soil feedback mechanisms within the northeastern margin of the Qinghai-Tibet Plateau (QTP). Utilizing Partial Least Squares Path Modeling (PLS-PM), we investigated the interrelationships among soil physicochemical properties, enzyme activities, species diversity, and community stability at varying post-fire recovery stages (5, 15, and 23 years). Results indicated that in the early recovery stages, rapid changes in soil properties such as decreased pH (*p* < 0.001) and increased nutrient availability facilitate the emergence of early successional species with high resource utilization traits. As the ecosystem evolved toward a climax community, the soil and vegetation exhibit increased stability. Furthermore, soil enzyme activities displayed dynamic patterns that corresponded with changes in soil nutrient content, directly influencing the regeneration and diversity of plant communities. Importantly, our study documented a transition in the influence of soil properties on community stability from direct positive effects in initial recovery phases to negative impacts in later stages, while indirect benefits accrue through increased species diversity and enzyme activity. Vegetation composition and structure changed dynamically with recovery time during community succession. Plant nutrient absorption and accumulation affected nutrient dynamics in the soil, influencing plant regeneration, distribution, and diversity. Our results underscore the complex interactions between soil and vegetation that drive the recovery dynamics post-wildfire, highlighting the resilience of forest ecosystems to fire disturbances. This study contributes to the understanding of post-fire recovery processes and offers valuable insights for the management and restoration of fire-affected forest ecosystems.

## Introduction

Fire, as a primary disturbance factor in terrestrial ecosystems, possesses a robust ability to regulate the ecological environment^1^. It significantly expedites the rates and turnover of nutrient elements in biogeochemical cycling, influencing the composition and structural characteristics of vegetation communities^2^, soil properties^3^, and multifunctional transformations in ecosystem nutrient cycling^4,5^. Consequently, it modifies the original plant-soil feedbacks within plant communities^6–9^. Post-fire, substantial alterations manifest in the biogeochemical and biostatistical characteristics of the plant-soil system^9,10^, potentially influencing or regulating interactions between vegetation, soil nutrients^11,12^, and feedback between aboveground and belowground biological communities^13^. The recovery of soil chemical stoichiometry significantly impacts vegetation community composition, diversity, nutrient cycling, and overall ecosystem functionality and stability^14,15^. Vegetation recovery is a crucial process for the restoration of ecosystem function due to its direct and indirect impacts on the physical, chemical, and biological properties of the soil^16^. Monitoring the recovery of plant-soil interactions in stable and disturbed ecosystems is essential for understanding ecosystem resilience^17,18^ and is a focal point in improving ecosystem management and enhancing ecosystem resilience^19–22^.

Plant-soil system plays a pivotal role in shaping long-term and large-scale patterns of plant growth, vegetation dynamics, and plant community diversity. Plants can modify the surrounding soil, consequently influencing plant growth^23^. Feedback interactions between plants and soil impact local biodiversity patterns worldwide^24,25^, thereby influencing plant succession and survival positively or negatively, leading to enduring effects on biodiversity and coexistence^26,27^. Fire can significantly alter plant-soil feedbacks by resetting or redirecting interactions between plants and soil biota. These feedback processes contribute to the temporal trajectory of plant community composition, playing a crucial role in spatial structuring and maintenance of species diversity^28–30^. Fire alters the biochemical characteristics of the soil, causing decreases in microbial biomass and soil enzyme activity, thereby decelerating soil biochemical reactions^31,32^. Plant-soil interactions persist in the post-fire environment, continually influencing plant growth rates and species composition^33,34^. Forest ecosystems with multi-layered vegetation and diverse plant communities are susceptible to high-intensity fires, influencing soil temperature throughout the soil profile and amplifying the impact on plant-soil feedbacks^35–38^. All these features and processes are intricately linked to the interactions between plants and soil, determining the recovery of soil quality and the composition and growth of vegetation^20,21^. Nevertheless, our comprehension of the interactions between these two ecosystems in the post-fire environment remains limited, and knowledge of the dynamic recovery mechanisms of plant-soil feedbacks in long-term post-fire recovery also remains inadequate^39^.

In undisturbed environments, a robust feedback dynamic exists between soil and vegetation, where the soil influences plant growth and composition, while vegetation, in turn, shapes soil properties through alterations in organic matter flux, nutrient return, and environmental conditions^40,41^. Several studies have explored post-fire plant-soil feedbacks in ecosystems. For instance, Keesstra et al.^42^ proposed that vegetation recovery could elevate soil water repellency in *Pinus halepensis* forests in Israel. Moya et al.^20^ observed that in *Pinus halepensis* forests in the southeastern Iberian Peninsula, vegetation renewal determined soil chemical properties, including pH, electrical conductivity, and various soil nutrients. López-Poma and Bautista^43^ and Mayor et al.^44^ identified a positive correlation between vegetation cover and soil enzyme activity in fire-prone shrublands in the eastern Iberian Peninsula. Additionally, the most substantial indirect impact of fire on the plant-soil system is the loss of litter and the alteration of soil organic matter, pH, and soil texture^45,46^. Post-fire, plant-soil feedbacks mediate by litter, particularly after severe burns involving the combustion of humus and organic layers, may lead to significant changes^38^. Despite the extensive attention devoted to the impact of fire on soil and vegetation in forest fire studies^47,48^, there is limited research on post-fire plant-soil interactions in the long-term recovery process, especially in the northeastern margin of the Qinghai-Tibet Plateau (QTP), where frequent fires occur. More comprehensive analyses are needed to ascertain whether consistent patterns of forest plant-soil feedbacks exist in different ecosystem types and regions after a fire.

This study aims to investigate plant-soil feedbacks in forest ecosystems on the northeastern margin of the QTP after wildfire. Specifically, we intend to employ the Partial Least Squares Path Modeling (PLS-PM) approach to analyze the interconnections among soil physicochemical properties, enzyme activities, species diversity, and community stability. Our goal is to assess the impact of time on vegetation restoration and its influence on soil properties in burned areas at different recovery stages (5, 15, and 23 years post-fire). We assume that the unburned areas exhibited similar soil and vegetation conditions before the wildfire. We hypothesize stronger plant-soil feedbacks in the short term after the wildfire compared to the mid-to-late recovery stages, as interspecific competition and environmental filtering within the plant-soil system intensify in the short term following disturbance. We anticipate that distinct plant-soil feedbacks will be influenced by the functional characteristics of vegetation. For instance, the faster growth and higher resource utilization of early successional species may result in more pronounced annual soil changes. The variation and relationships of soil quality indicators during short, medium, and long-term post-fire recovery are expected to be linked to vegetation community recovery.

## Results

### Effects of different restoration years on species diversity

**Figure 1:**
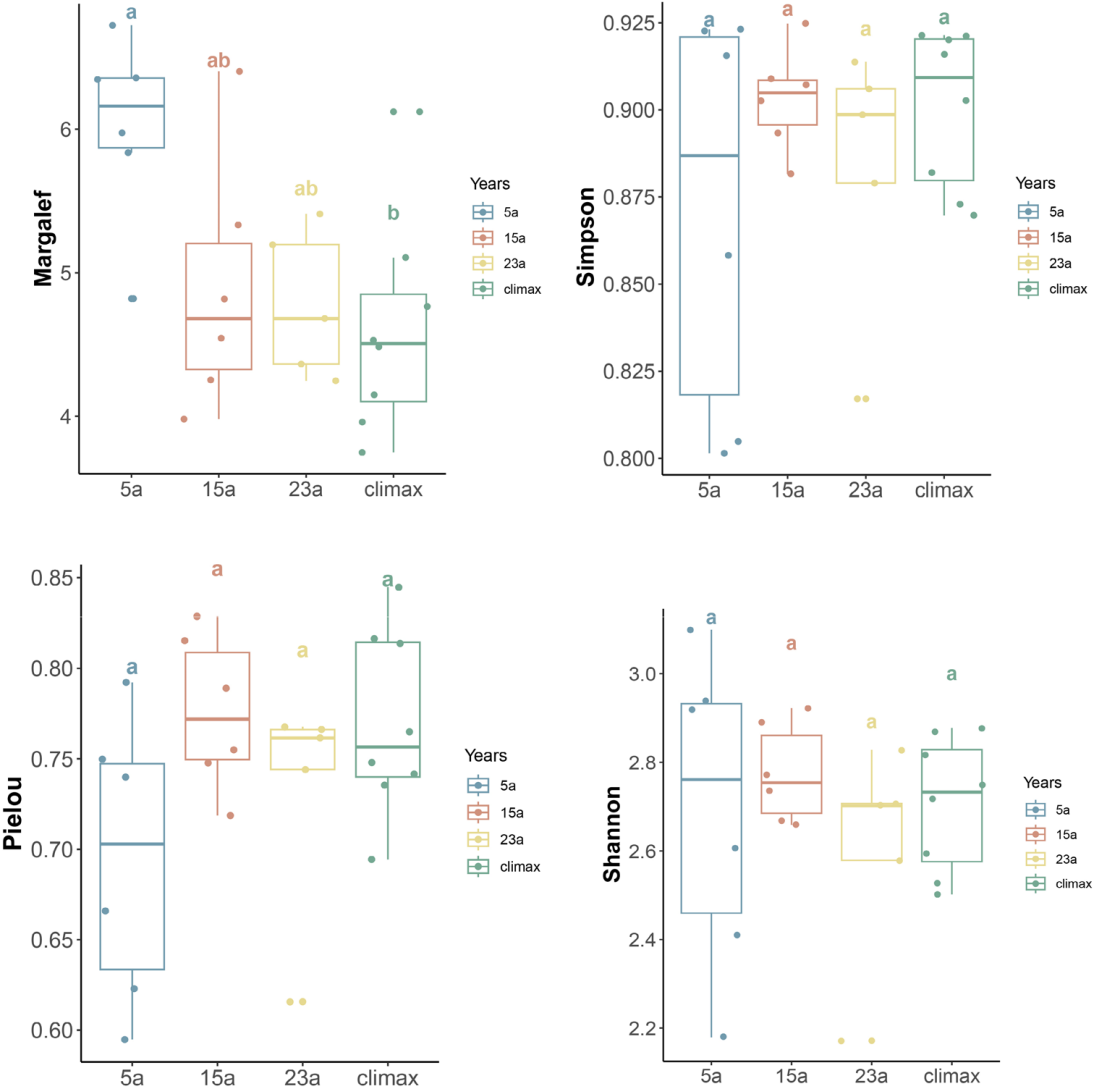
Species diversity characteristics among the different periods. Different lowercase letters (a, b, and ab) indicate significant differences (*p* < 0.05) within each variable among the different restoration periods. Diverse colors signify distinct stages of recovery. 5a, 15a, 23a: restoration years for 5, 15 and 23 years, respectively; Climax: climax community.

In this study, it was found that the vegetation recovery sequence supported 174 species of seed plants, which represented 49 families and 108 genera (Table S1). The species diversity showed that both Margalef and Shannon indices of all sampled communities exhibited a decrease with increasing restoration duration, with Margalef index displaying significant variation in this trend (*p* < 0.05). Conversely, Pielou index and Simpson index increased as restoration duration extended (Fig. 1). The analysis of four indices of species diversity revealed that there was a notable occurrence of steep increases or decreases in plant species diversity indices between the 5a and 15a sites, with the trends becoming more gradual over time.

### Soil chemical composition

**Figure 2:**
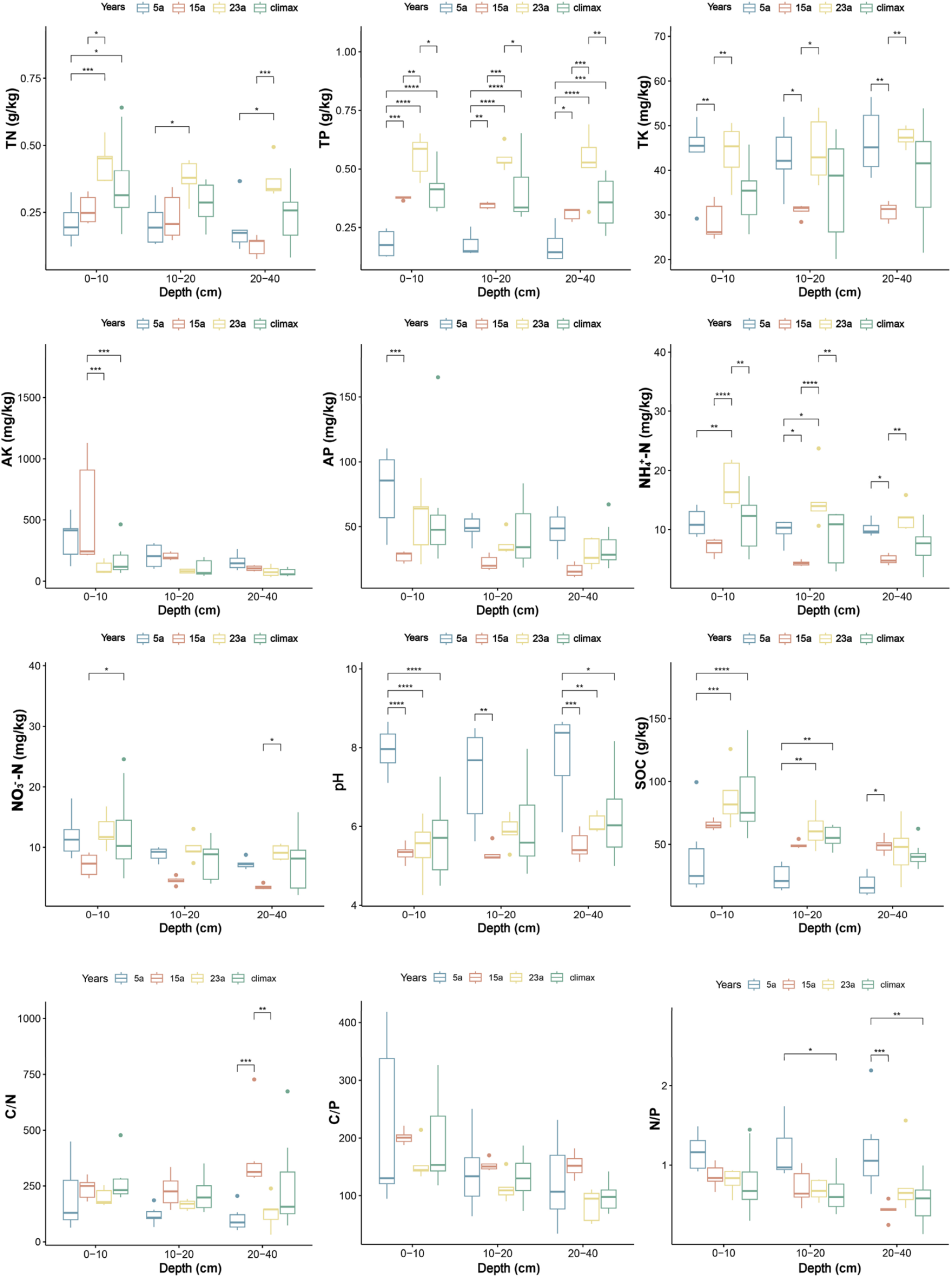
The changes in soil chemical properties at different recovery stages (*p ≤ 0.05; **p ≤ 0.01; ***p ≤ 0.001; and****p ≤ 0.0001, respectively).

Examining the results (Fig. 2), significant differences were observed in the soil stoichiometric characteristics across various recovery stages. With increasing recovery time, the TN (total nitrogen) content, TP (total phosphorus) content, and C/N (carbon–nitrogen ratio) exhibited an increasing trend, while AK (available potassium), AP (available phosphorus), C/P (carbon-phosphorus ratio), and N/P (nitrogen-phosphorus ratio) content showed a decreasing trend. Conversely, TK (total potassium), NH_4_^+^-N (ammonium nitrogen), NO_3_^−^-N (nitrate nitrogen), and pH (potential of hydrogen) content decreased first then increased. TP content showed significant differences in all recovery stages and across all soil layers, emerging as the most pronounced chemical component with varying dynamics in the soil. In the 0–10 cm soil layer, TN content showed a significant increase over recovery time, with non-significant differences between 5 and 15a but significant differences between 5 and 23a. TK content exhibited a significant decrease between 5 and 15a in all soil layers, followed by a significant increase between 15 and 23a. The sharp decrease and subsequent increase of NH_4_^+^-N content with increasing recovery time resulted in significant differences across all soil layers. The pH values of each soil layer were alkaline in stage 5a, significantly higher than in the other recovery stages, and gradually stabilized to an acidic state in the later stages of recovery. As recovery time increases, AK, AP, NO_3_^−^-N, C/N, and C/P exhibited minimal or non-significant differences across different soil layers. Soil organic carbon (SOC) showed a significant increase with increasing recovery time, with the most pronounced changes occurring predominantly between 5 and 15a. These results indicated that the significant changes in most soil physicochemical properties after the wildfire were concentrated in the shallow soil layers (0–10 cm) during the post-fire recovery stages.

### Soil enzyme activity

**Figure 3:**
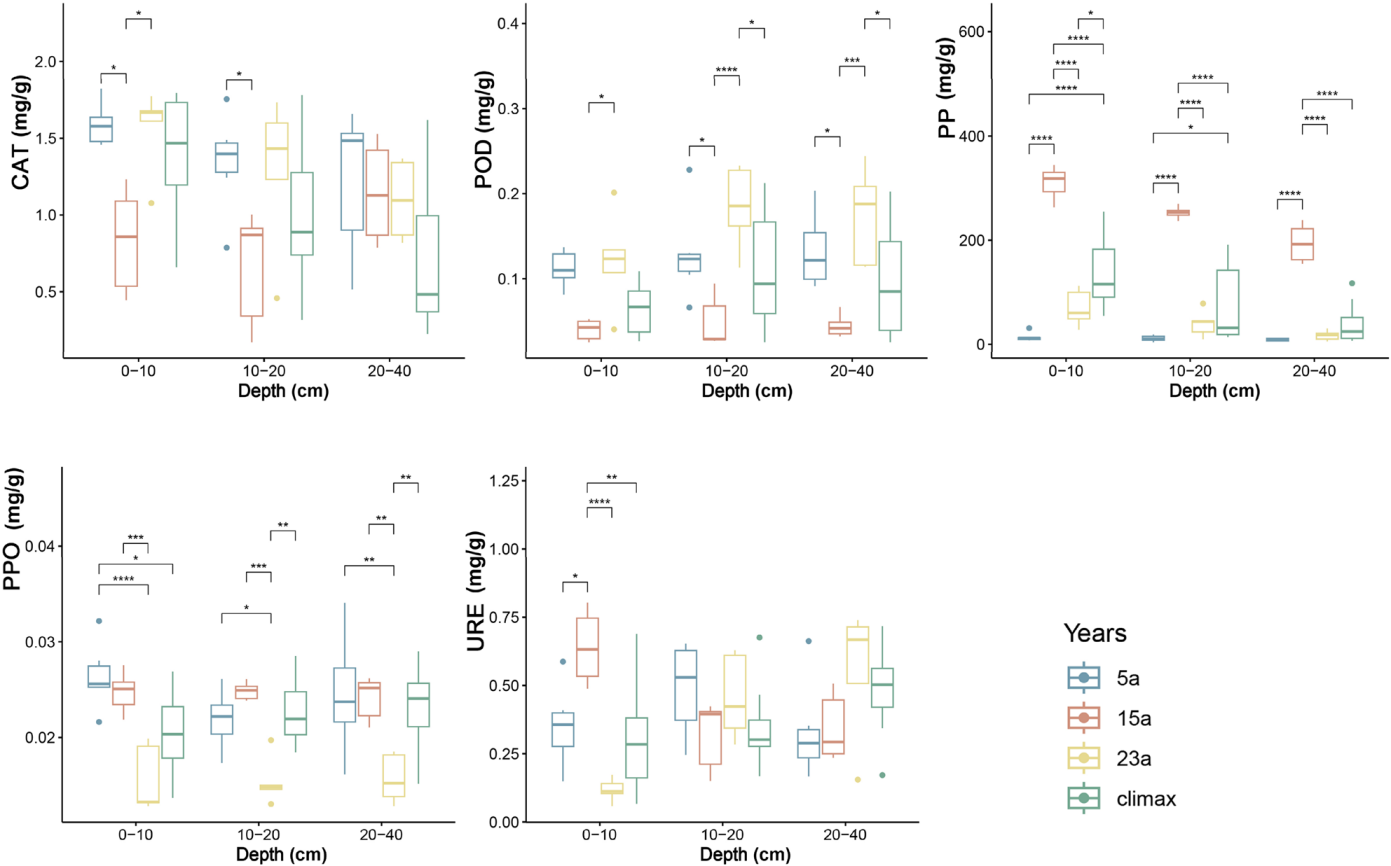
Soil enzyme activity in different recovery stages. CAT, POD, PP, PPO, URE correspondingly denote the abbreviations for Catalase, Peroxidase, Phosphatase, Polyphenol oxidase, and Urease (*p ≤ 0.05; **p ≤ 0.01; ***p ≤ 0.001; and ****p ≤ 0.0001, respectively).

As depicted in Fig. 3, there were significant differences in soil enzyme activities across various recovery stages. The overall trend for catalase (CAT) and peroxidase (POD) activities showed an initial decrease followed by an increase, while phosphatase (PP) and urease (URE) activities increased first then decreased. The significant variations in CAT activity occurred between the 5a and 15a stages, with diminishing differences as soil depth increases. POD activity displayed more pronounced differences in deep soil layers compared to shallow soil layers. PP activity exhibited highly significant variations with recovery time, and significant differences persisted in all layers of soil. Polyphenol oxidase (PPO) activity showed a significant decrease with increasing recovery time after the wildfire, but in the later stages of recovery, it was lower than the soil activity in the climax community. URE activity only showed significant changes in the 0–10 cm soil layer over recovery time. These findings underscored the dynamic nature of soil enzyme activities during the post-fire recovery stages, highlighting nuanced responses to different enzyme types and soil depths.

### Relationships among soil chemical properties, enzyme activities and species diversity

**Figure 4:**
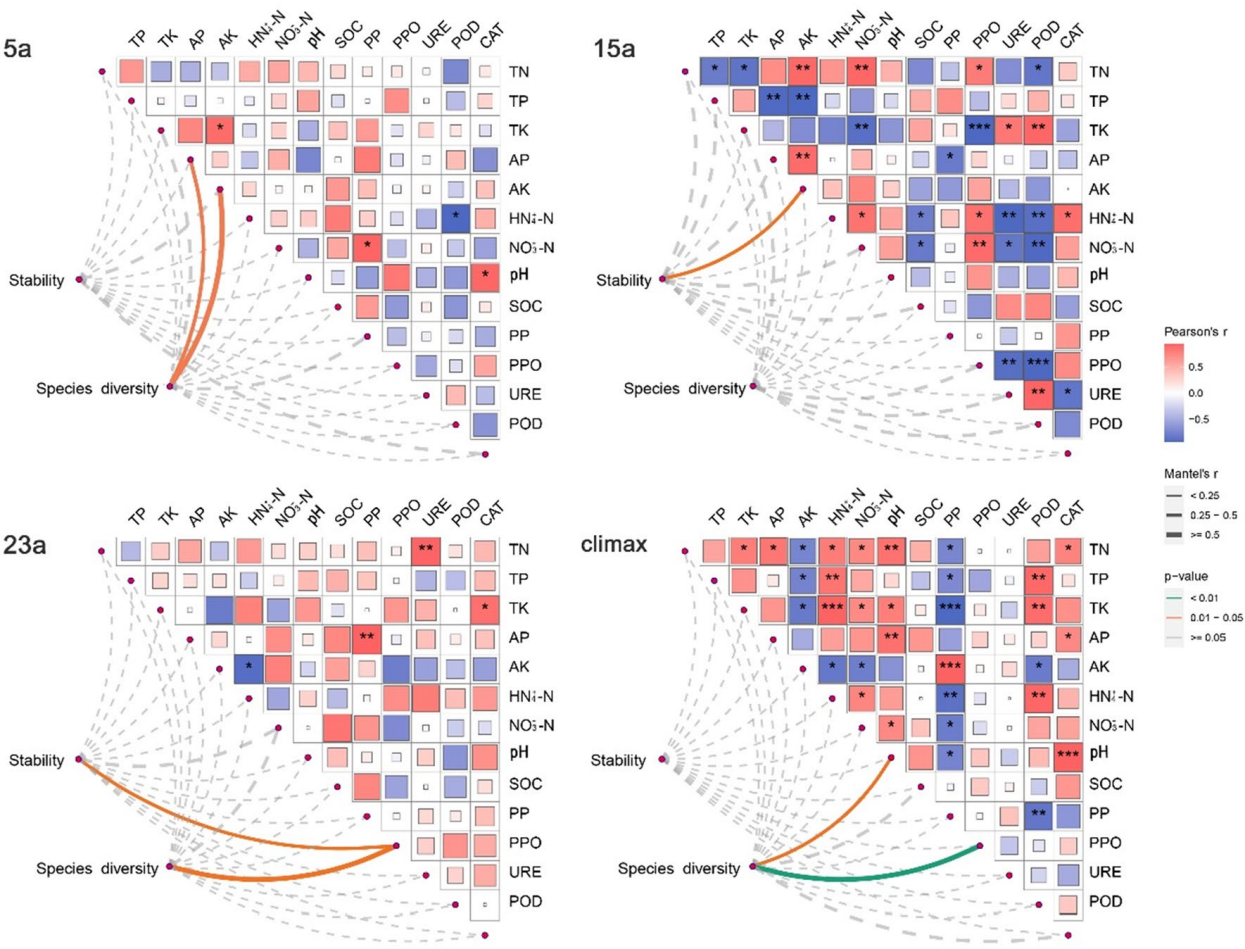
The mantel test on the relationship between species diversity and soil characteristics. Legend: 0 represents no correlation, 1 represents the greatest positive correlation, and − 1 represents the greatest negative correlation. The proportion of the square filled area represents the absolute value of the correlation. The value of the correlation is indicated by the shaded color and saturation. *CAT*, catalase, *POD* peroxidase; *URE* urease, *PPO* polyphenol oxidase, *PP* phosphatase. *p < 0.05; **p < 0.01; ***p < 0.001.

The results revealed differential changes in soil chemical properties, enzyme activities, and species diversity across various recovery stages following the wildfire (Fig. 4).

During the early recovery phase (5a), a significant positive correlation was observed between pH and CAT, as well as between NO_3_^-^-N and PP (*p* < 0.05). Conversely, NH_4_^+^-N exhibited a significant negative correlation with POD (*p* < 0.05). Mantel tests indicated a significant positive correlation between species diversity and AP, AK, and C/N ratio (*p* < 0.05), while community stability showed a significant positive correlation with AP and AK (*p* < 0.05) (Table S2).

In the 15a recovery stage, a significant positive correlation was observed between TN and PPO (*p* < 0.05), while TN exhibited a significant negative correlation with POD (*p* < 0.05). TK showed a significant positive correlation with POD and URE (*p* < 0.05, *p* < 0.01), and a highly significant negative correlation with PPO (*p* < 0.001). Additionally, AP exhibited a significant negative correlation with PP (*p* < 0.05). NH_4_^+^-N demonstrated a significant positive correlation with CAT and PPO (*p* < 0.05), while it showed a significant negative correlation with POD and URE (*p* < 0.01). NO_3_^-^-N displayed a significant positive correlation with PPO (*p* < 0.01), and a significant negative correlation with POD and URE (*p* < 0.01, *p* < 0.05). Mantel tests indicated that, during this stage, only community stability had a significant positive correlation with AK (*p* < 0.05) (Table S3).

In the 23a recovery stage, a significant positive correlation was observed between TN and URE (*p* < 0.01), TK and CAT (*p* < 0.05), and AP and PP (*p* < 0.01). Both community stability and species diversity exhibited significant positive correlations with PPO (*p* < 0.05) (Table S4).

For climax community, significant differences in soil properties and enzyme activities were primarily associated with CAT, POD, and PP. Notably, TN, AP, pH, and N/P exhibited significant positive correlations with CAT (*p* < 0.05, *p* < 0.001). POD showed significant positive correlations with TP, TK, and NH_4_^+^-N (*p* < 0.01), while displaying significant negative correlations with AK and C/N (*p* < 0.01, *p* < 0.05). PP exhibited significant negative correlations with TN, TP, TK, NH_4_^+^-N, NO_3_^-^-N, and pH (*p* < 0.05, *p* < 0.01, *p* < 0.001), along with significant positive correlations with AK and C/N (*p* < 0.001, *p* < 0.05). Mantel tests revealed significant positive correlations between species diversity and pH, C/P, and PPO (*p* < 0.05, *p* < 0.01) (Table S5). These findings underscored the intricate relationships between soil characteristics, enzyme activities, and species diversity during different recovery stages post-fire, providing valuable insights into the ecological dynamics of post-fire ecosystems.

### PLS-PM for community recovery

**Figure 5:**
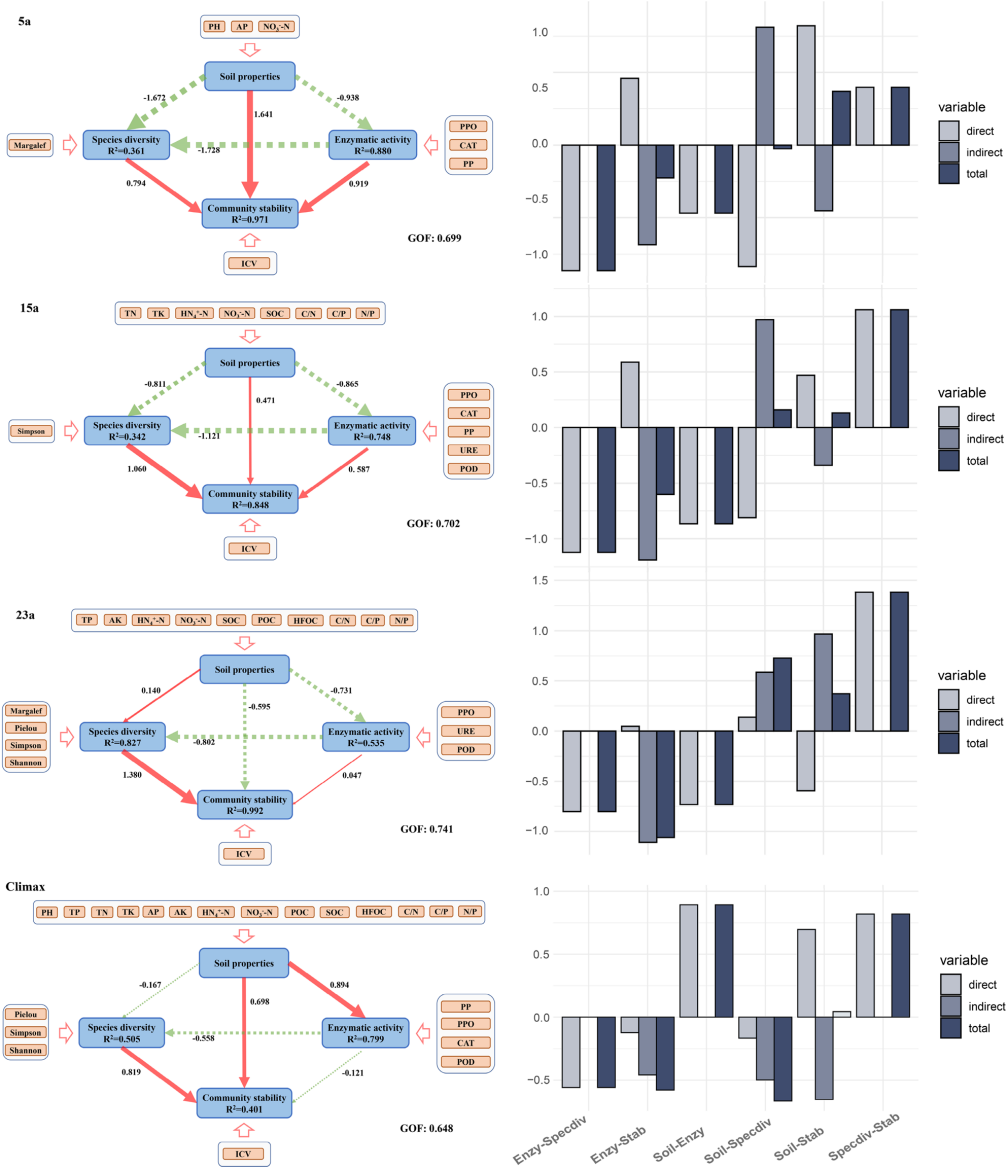
PLS-PM of 5a, 15a, 23a, and climax community, and direct, indirect, and total effect between variables in the inner model of different factors. Blue rectangles and orange rectangle indicate latent and manifest variables, respectively. Unidirectional cause-total effect between latent variables is shown as an arrow with path coefficient (red and green arrows indicate positive and negative effects, and the thickness of the arrow line represents the size of the path coefficient in the model, respectively). The R^2^ indicates the determination coefficient of endogenous latent variables (Coefficients of determination R^2^): ≥ 0.600, better; 0.600–0.300, moderate; < 0.300, poor. GOF > 0.600: indicating that the model has a good fitting degree. Enzy, enzyme activity; Stab, community stability; Specdiv, species diversity; Soil, soil characteristics.

In order to comprehensively explore the variation characteristics of vegetation and soil mixtures at different recovery stages after wildfires, PLS-PM was constructed (Fig. 5, Table S7).

The results indicated that, with increasing recovery years, the direct impact of soil physicochemical properties on community stability gradually diminished (Fig. 5, Table S7). In the 5a stage, soil physicochemical properties exhibited a significant direct positive impact on community stability (direct effect value (DEV): 1.641), while in the 23a stage, they demonstrated a negative effect on community stability (DEV: − 0.595). Although the direct effect of soil physicochemical properties on community stability was gradually decreasing, the indirect effect was increasing over the recovery years (indirect values (IEV): − 0.903, − 0.339, 0.968, respectively). Simultaneously, the negative impact of soil physicochemical properties on species diversity and enzyme activity weakened over time. The direct impact on species diversity turned positive in the 23a stage (DEV: 0.140), and within the climax community, soil physicochemical properties significantly positively influenced enzyme activity (DEV: 0.894). Furthermore, the indirect impact of soil physicochemical properties on species diversity was gradually diminishing (IEV: 1.621, 0.969, 0.587), while enzyme activity was entirely influenced by the direct impact of soil physicochemical properties.

At different recovery phases (Fig. 5, Table S7), the direct impact of enzyme activity on community stability is positively correlated, but this positive influence gradually diminished (DEV: 0.919, 0.587, 0.047), turning negative within the climax community (DEV: − 0.121). Additionally, the indirect impact of enzyme activity on community stability exhibited a significant negative influence, which gradually weakened over time, reaching the climax community stage (IEV: − 1.372, − 1.189, − 1.107, − 0.457). The direct impact of enzyme activity on species diversity was consistently negative at all stages, and this negative influence weakened over time (DEV: − 1.728, − 1.121, − 0.802, − 0.558). As recovery time progresses, until the climax community stage, species diversity consistently exhibited a significant positive impact on stability (DEV: 0.794, 1.060, 1.380, 0.819). In comparison to the climax community, soil physicochemical properties, enzyme activity, and species diversity gradually underwent succession toward the original native community.

## Discussion

### Change characteristics of plant-soil system with restoration time

The research indicated that the plant-soil systems of the burned areas in the northeastern margin of the QTP varied in different recovery stages. Regarding species diversity, the Margalef index of the community increased significantly (*p* < 0.01) during the early stages of post-fire succession compared to the climax community, while the Shannon, Pielou, and Simpson indices did not differ significantly from pre-fire levels. These indices gradually approached the levels of the climax community as recovery time progressed (Fig. 1). The driving factors during the early stages of succession might be mainly influenced by surface exposure, ample sunlight, and insufficient soil nutrients^49^. The significant increase in the Margalef index during the early stages of recovery could result from the substantial loss of species caused by the wildfire. Plants with characteristics such as ease of reproduction, photophilicity, and nutrient tolerance quickly invaded and spread in the burned areas, establishing and proliferating continuously. This increased the number and richness of species in the community. The unique ecological characteristics of these species enabled them to utilize the nutrients provided by ash^47^, making them pioneer or even dominant species in the burned areas^50^. Examples include herbaceous plants such as *Carex crebra, Ligularia botryodes, Veronica polita, Saussurea amara*, and others (Table S1), which were more likely to establish themselves in the burned regions than other plants^51^. As recovery time increased into the mid to late stages of community succession, plant species with lower adaptability and weaker competitiveness decreased through interspecific competition, dispersal, and environmental filtering and selection. This increased plant species with stronger adaptability and broader ecological niches, including the emergence of more stable or advanced species such as trees and evergreen plants, enhancing the stability of community structure^52^. This maintained the diversity and stability of the ecosystem^53^.

The research findings indicated that the soil TP and TN content in the burned area was significantly lower than in the unburned area (*p* < 0.001, *p* < 0.05) during the early stages of post-fire succession (Fig. 2). This might result from the combustion of organic matter and the volatilization of nutrient elements in the soil due to the fire, releasing a substantial amount of phosphorus and nitrogen. The TP and TN levels in the soil decreased as smoke dispersed or rain leached. TN and TP gradually increased with the progression of succession. This trend was attributed to the increasing input of litter during the recovery of vegetation over time. Accumulation of soil organic matter might enhance microbial activity, accelerating the decomposition and mineralization of organic matter in root residues and litter layers, thereby increasing soil nitrogen and phosphorus content^54^, in line with previous research findings^55^. In the 23-year stage, the TP and TN content in all soil layers were higher than in the climax community (Fig. 2). This might be due to changes in vegetation composition and structure during the later stages of community succession. Plants with higher nutrient absorption and accumulation capabilities might lead to higher levels of P and N in the soil than climax communities^56^. Compared to the climax community, the AP and AK content in burned soils was higher during the early stages of recovery. This might result from the combustion of organic matter and plant residues during the fire, releasing nutrients absorbed and stored in plant tissues during growth and metabolism, which leads to relatively higher AP and AK content in the soil^57^. The content of AP and AK gradually decreased as recovery time extended. This might be attributed to the enhanced ability of plant roots to absorb AP and AK, causing their gradual reduction, consistent with other research findings^58–60^. Additionally, there was no significant difference in NO_3_^-^-N and NH_4_^+^-N content compared to the climax community during the early stages of post-fire succession, and a trend of initial decrease followed by an increase was observed with the progression of succession, ultimately stabilizing. This trend might be related to the burning of vegetation and organic matter during the fire, releasing nutrient substances (including NO_3_^-^-N and NH_4_^+^-N) into the soil^61^. Changes in vegetation types with different nitrogen absorption and release characteristics might contribute to the dynamic fluctuations of NO_3_^−^-N and NH_4_^+^-N with ongoing succession^62^.

During the early stages of recovery, the hydrolysis of alkaline cations in ash produced by the fire resulted in alkaline soil pH^63,64^. The pH decreased significantly and turned acidic as recovery time extended. This might be due to enhanced root respiration in community plants, increased release of organic acids from plant roots, and possibly decomposition of dead plant material by microorganisms^65^. Different species might exhibit varying degrees of tolerance and adaptability to soil pH. Plants with stronger adaptability could survive under different pH conditions, contributing to the stability of the community^66^. The SOC content in the burned area decreased significantly immediately after the fire but increased gradually over time. This might result from the gradual accumulation of contributions from plants and other organic matter during the rebuilding and succession of vegetation^67^. New organic matter inputs might include root exudates, litter, dead plants, and microorganisms, contributing to the gradual increase in soil organic carbon^68^. The interaction between soil and vegetation was dynamic and continually improved soil conditions to provide suitable habitats for various plants^69^.

Soil enzyme activity is closely correlated with the species composition in ecosystems^70^. The results of this study (Fig. 3) showed that fire significantly altered soil enzyme activity^61^, as indicated by the significant decrease in PP (*p* < 0.0001) and increase in PPO (*p* < 0.0001) during the early stages of post-fire succession compared to the climax community. The initial increase in pH and nutrient loss after fire could reduce PP activity, which would limit the availability of inorganic phosphorus in the soil and affect plant growth^71^. Simultaneously, fire could enhance the content of polyphenolic substances in the soil, which are secondary metabolites of some fire-resistant plants^72^. These substances stimulated PPO activity, which increased the production of quinone compounds in the soil and improved soil antioxidant and antibacterial capabilities^73^. Conversely, enzymes such as CAT, POD, and URE recovered to pre-fire levels relatively quickly, suggesting that fire had weak or transient effects on these enzymes or that they were more accessible in the post-fire soil^74^. CAT and POD are enzymes involved in soil organic matter decomposition and redox reactions^75^. In the early stages of post-fire recovery, the lower soil organic matter and redox potential resulted in decreased CAT and POD activity^76^. However, as soil organic matter and redox potential gradually recovered or increased over time, CAT and POD activity also increased^71^. This could be attributed to plant regeneration and microbial recolonization, which provided organic matter and oxygen to the soil and enhanced its redox potential^77^. CAT and POD activity decreased with increasing soil depth, possibly due to changes in soil temperature and moisture, which affect the stability and activity of soil enzymes^78^. PP and URE are enzymes related to P and N cycling^77^. Fire can cause the volatilization or migration of P and N in the soil, changing the content and form of P and N in the soil^61^. In the early stages of post-fire recovery, the low content of inorganic P and N in the soil (Fig. 2) lead to increased PP and URE activity. This could be because PP and URE can hydrolyze organic P and N compounds in the soil, releasing inorganic P and N to meet the demands of plants and microorganisms^79^. Over time, the content of inorganic P and N in the soil gradually recovered or increased, leading to decreased PP and URE activity. This change could be associated with plant uptake and microbial nitrogen fixation and mineralization, which reduced the availability of soil P and N by consuming or transforming inorganic P and N in the soil^80^. PPO is an enzyme that oxidizes polyphenolic substances in the soil, producing quinone compounds with antioxidant and antibacterial properties^81^. Fire increased the content of polyphenolic substances in the soil, as these substances are secondary metabolites of some fire-resistant plants^82,83^. In the early stages of post-fire recovery, the high content of polyphenolic substances in the soil led to increased PPO activity. This was because PPO used polyphenolic substances as substrates for oxidation reactions, enhancing the redox potential of the soil^81^. Over time, the content of polyphenolic substances in the soil gradually decreased, resulting in a decrease in PPO activity. This change could be related to plant decomposition and microbial degradation, as plants and microorganisms can consume or transform polyphenolic substances in the soil, reducing the effectiveness of soil polyphenolic substances^84^. PPO activity significantly decreased with the increase in post-fire recovery time but remained lower than the soil activity in climax communities during the late recovery period. This may be related to soil organic matter and redox potential, as these factors can affect the expression and activation of PPO^81^.

Fire-induced changes in soil enzyme activity can affect the structure and composition of plant communities. The reduction of inorganic phosphorus in the soil after fire can constrain the growth of some phosphorus-demanding plants, reducing their dominance^85^. Conversely, the increase in quinone compounds in the soil after fire can suppress the growth of some plants sensitive to polyphenolic substances, lowering their dominance^86^. Thus, the diversity of plant communities may decline during the early stages of post-fire recovery, and plants with high tolerance to phosphorus and polyphenolic substances may prevail^73^. Over time, soil enzyme activity and plant communities may gradually return to pre-fire conditions or attain a new equilibrium (Fig. 5).

### Relationship between plant and soil physicochemical properties and soil enzyme activities in plant-soil system

Wildfires can change soil chemical properties, affecting the activity and types of soil enzymes, which influence soil nutrient cycling and transformation, as well as the regeneration, distribution, and diversity of plants. In the early stages of recovery after a fire (5a), the soil pH increased, which increased CAT activity^87^. This protected soil microorganisms from hydrogen peroxide toxicity^88^. Meanwhile, the soil NO_3_^−^-N content increased, positively correlated with PP activity, as PP could break down organic phosphorus, providing an available phosphorus source^88,89^. However, NH_4_^+^-N content decreased, negatively correlated with POD activity, as POD could break down lignin, providing an available carbon source^90^. Moreover, the Mantel test showed a positive correlation between species diversity and AP, AK, and C/N, indicating that plants had a higher demand for phosphorus and potassium, and a lower demand for nitrogen^91^. Community stability s positively correlated with AP and AK, suggesting that effective phosphorus and potassium in the soil could maintain community stability^92^. In the 15a post-fire recovery stage, soil TN content increased, positively correlated with PPO activity and negatively correlated with POD activity. This is because PPO can oxidize polyphenolic substances, providing an available nitrogen source, while POD had the opposite effect^93^. Soil TK content increased, positively correlated with POD and URE activity, and negatively correlated with PPO activity. This is because POD and URE can break down organic nitrogen, providing an available potassium source, while PPO had the opposite effect^93^. Soil AP content decreased, negatively correlated with PP activity, because soil phosphorus content inhibited PP activity^57^. Soil NO_3_^−^-N and NH_4_^+^-N content increased, positively correlated with CAT and PPO activity, and negatively correlated with POD and URE activity. This is because CAT and PPO can enhance soil nitrogen mineralization, while POD and URE could enhance soil nitrogen fixation^73^. Additionally, the Mantel test showed (Fig. 4) a significant positive correlation between community stability and AK, indicating that effective potassium in the soil is a key factor influencing plant community stability in the mid-term recovery.

In the 23a post-fire recovery stage, soil TN, TK, and AP content increased, positively correlated with URE, CAT, and PP activity. This accelerated the cycling and transformation of nitrogen, potassium, and phosphorus in the soil, enhancing the availability of soil nutrients^94^. Community stability and species diversity were both positively correlated with PPO activity, indicating that PPO could promote plant utilization of polyphenolic substances, increasing plant adaptability and diversity^95^. In the climax community, the significant differences in soil properties and enzyme activity are mainly related to CAT, POD, and PP. These three enzymes played crucial roles in the cycling and transformation of soil carbon, nitrogen, and phosphorus^96^. Specifically, TN, AP, pH, and N/P were positively correlated with CAT activity, indicating that CAT could promote soil nitrogen and phosphorus mineralization, enhancing soil acidity and nitrogen-phosphorus ratio^91^. POD is positively correlated with TP, TK, and NH_4_^+^-N, but negatively correlated with AK and C/N, because POD could promote soil phosphorus mineralization, increasing the ammonium nitrogen content and decreasing the available potassium content and carbon–nitrogen ratio in the soil^97^. PP is negatively correlated with TN, TP, TK, NO_3_^-^-N, NH_4_^+^-N, and pH, but positively correlated with AK and C/N, because PP could promote phosphorus fixation in the soil, reducing nitrogen, potassium, acidity, and nitrogen-phosphorus ratio, and increasing available potassium content and carbon–nitrogen ratio in the soil^98^. Additionally, the Mantel test showed that species diversity is positively correlated with pH, C/P, and PPO activity, suggesting a certain adaptability of plants to soil acidity, carbon-phosphorus ratio, and polyphenolic substances. The impact of wildfires on soil chemical properties, enzyme activity, and species diversity was complex, with different characteristics and patterns in different recovery stages.

### Dynamic recovery of maintaining community stability of plant-soil system after fire

Regional-scale environmental factors, such as soil nutrients, are determinants of plant community distribution^99,100^. Environmental factors affect the diversity-community stability relationship in various ways, such as positive facilitation^101^, unimodal effects^102^, negative constraints^56^, or no significant influence. However, these relationships are poorly understood in post-severe-fire vegetation recovery areas. Our study showed that soil factors influenced community stability directly and indirectly by regulating species diversity. Nutrient-rich soils were crucial for plant growth and development and shaped the composition and diversity of plant communities^103^.

After a fire, the direct impact of soil physicochemical properties on community stability shifted from positive to negative, while the indirect impact changed from negative to positive (Fig. 5). This showed that fire altered soil organic matter, nutrient, and pH levels, affecting plant growth and competitive abilities^75^. Over time, the soil and plants adapted to the new environmental conditions, increasing community stability^104^. The direct effect of soil physicochemical properties on species diversity and enzyme activity became positive, while the indirect effect weakened^105^. This indicated that fire reduced soil organic matter and nutrient content, lowering species diversity and enzyme activity. As time passed, soil organic matter and nutrient content increased, enhancing species diversity and enzyme activity. Meanwhile, the indirect effect of soil physicochemical properties on species diversity and enzyme activity was mainly mediated by community stability. As community stability increased, this mediation decreased.

In the process of community restoration, the direct impact of enzyme activity on community stability gradually diminished, while the indirect impact gradually intensified (Fig. 5). This showed that fire damaged soil enzyme activity by high temperatures, reducing community stability^106^. Over time, soil enzyme activity recovered but was also affected by soil physicochemical properties and species diversity, increasing the indirect effect on community stability^106^. The direct effect of enzyme activity on species diversity was consistently negative but weakened. This indicated that fire lowered soil enzyme activity, which negatively affected species diversity, possibly by limiting the cycling and availability of soil nutrients, which constrained species growth and reproduction^79^. As time passed, soil enzyme activity recovered, but the negative effect on species diversity remained. This could be because soil enzyme activity was related to competition and inhibition by soil microorganisms, which influenced species diversity^107^.

With the increaseing recovery time, the direct effect of species diversity on community stability was consistently positive and strengthened (Fig. 5). This suggested that fire increased species diversity, which enhanced community stability, likely by increasing functional diversity and redundancy, which enhanced the community’s adaptability and resistance to environmental changes^108,109^. Over time, the positive effect of species diversity on community stability strengthened. This could be attributed to the increased diversity enhancing the complementarity and synergistic interactions within the community, which elevated community productivity and efficiency^110,111^. Therefore, as the recovery period extended, plant communities underwent positive succession, resulting in a continuous enhancement of community stability.

## Materials and Methods

### Study area

**Figure 6:**
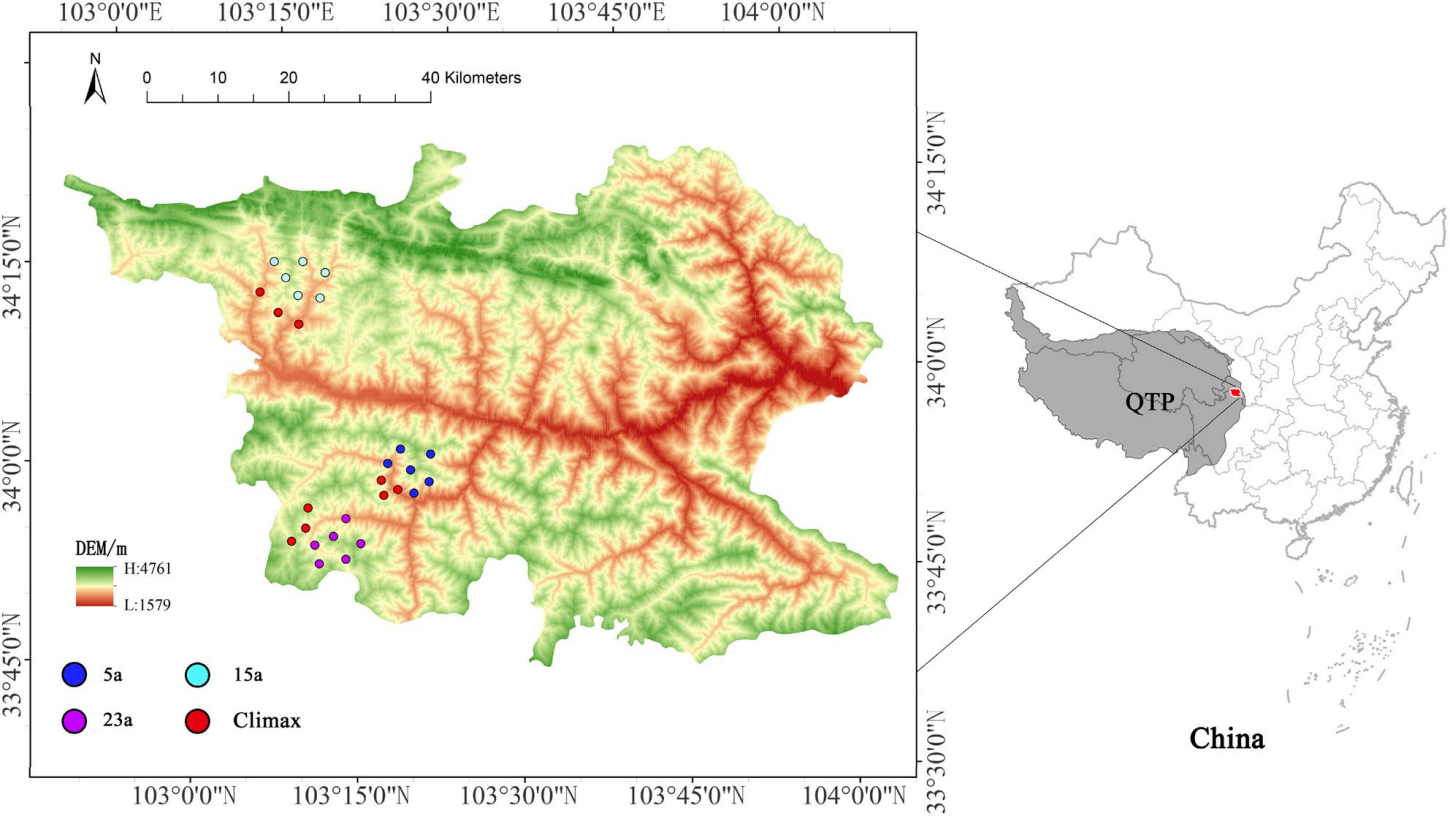
Spatial distribution of the study sites, created with ArcGIS 10.8 software (www.esri.com).

The study area is situated in the forest region of Diebu County, Gansu Province, located on the northeastern margin of the QTP within the high-mountain canyons of the Bailong River Basin. It belongs to the mountainous vegetation region of the Gannan Plateau, serving as a crucial watershed between the Yangtze River Basin and the Yellow River Basin^52^. The sampling sites span from 33°49′18.84'' to 34°38′38'' N and from 103°12′48.69'' to 103°21′22.14'' E (Fig. 6). The predominant native plant community is primarily composed of *Picea asperata-Abies fargesii* forest, indicative of a transitional climate between the temperate zone and the alpine region on the northeastern margin of the QTP. The region experiences a mean annual temperature of 7.5 °C, an average annual precipitation of 568 mm, an annual average evaporation of 1444.2 mm, an annual average sunshine duration of 2308.0 h, and an annual relative humidity ranging from 52 to 76%. The average frost-free period is approximately 134 days. Notably, due to local topography, the climate displays discernible vertical variations^112^.

Over the years, this region has experienced frequent forest wildfires. To explore the relationship between vegetation and soil properties across different recovery stages post-wildfire, we utilized fire records from the local forestry bureau and conducted field surveys on plots that had suffered similar intensities of fire damage, specifically choosing sites with low environmental heterogeneity. Employing the "space-for-time substitution" approach, we selected sites that experienced severe wildfires in 1997, 2005, and 2015 and subsequently suffered no recurring fires (Fig. S1). These sampling sites were characterized by the total destruction of ground-level biota and the ecosystem at large, leaving only standing or fallen dead trees. After these severe fire events, local authorities promptly enacted enclosure measures to facilitate the natural regeneration of biota and the ecosystem at these locations. This methodology allows for a systematic investigation into ecological recovery processes over time within a consistent geographical framework (Table S6).

### Experimental design and data collection

In the period from July to August in 2021, we conducted field surveys of plant communities and soil sampling. Based on the complexity of plant species composition and structure at different successional stages and in consideration of the topographical characteristics of the burned areas, we established 6, 6, and 5 quadrat plots measuring 20 × 20 m in the 5a, 15a, and 23a post-fire recovery stages, respectively. Additionally, in the climax community (primitive forest) adjacent to the 5a, 15a and 23a burned areas, three quadrat plots with an area of 20 × 20 m were set up respectively. We recorded information such as the altitude, slope, aspect, and geographic coordinates of each sample site. Within the tree quadrat plots, we employed the "five-point method" to set up 5 subplots measuring 5 × 5 m in the east, south, west, north, and center of each plot, respectively, to investigate shrub characteristics, including species, number of individuals (or clumps), average tree height, basal diameter, crown width, and canopy cover. In each shrub subplot, we further established a 1 m × 1 m herb subplot to investigate herb characteristics, including species, number of individuals (or clumps), average height, and canopy cover. After completing the vegetation survey, we measured the thickness of the litter in every herb plot, collected the litter in the quadrat, and packed the litter in a self-sealing bag, weighed it, and then brought it back to the laboratory. Then, we collected soil samples from each herb subplot at depths of 0–10 cm, 10–20 cm, and 20–40 cm using soil augers. Five samples from the same soil layer within each site were mixed evenly to obtain a composite soil sample, which was then sealed in self-sealing Polyethylene bags and labeled for preservation after removing debris. Subsequently, the preserved soil samples were brought back to the laboratory for timely air-drying. After air-drying, the samples were sieved through a 100-mesh sieve for soil physical and chemical properties.

### Soil properties

The soil physicochemical analyses followed the methodology outlined by Lu^113^. After digesting the samples with 0.5 mol·L^−1^ potassium dichromate and sulfuric acid, the determination of SOC content was conducted using the 0.3 mol·L^−1^ ferrous sulfate titration method. Soil pH was measured in a 1:5 soil–water suspension using a pH meter (Mettler-Toledo S220, Switzerland). The determination of TN content was carried out using the Kjeldahl method^114^. TP content was measured using the molybdenum antimony blue colorimetric method after digestion with sodium hydroxide. TK content was determined using a flame photometer (BWB-XP, BWB Technologies, UK) after mixed digestion with concentrated sulfuric acid and perchloric acid. NH_4_^+^-N and NO_3_^-^-N were extracted with 2 mol·L^−1^ KCl and quantified using a continuous segmented flow analyzer (SEAL-AA3, SEAL Analytical Limited, UK). AP was determined using the molybdenum antimony blue colorimetric method after extraction with 0.5 mol·L^−1^ sodium bicarbonate for neutral and alkaline soils, and 0.03 mol·L^−1^ ammonium fluoride and 0.025 mol·L^−1^ hydrochloric acid for acidic soils. AK was extracted with 1 mol·L^−1^ ammonium acetate (NH_4_OAc) and determined using a flame spectrophotometer (BWB-XP, BWB Technologies, UK).

### Soil enzyme activities

The activity of URE was determined using the phenol-sodium hypochlorite colorimetric method, and its activity was expressed as the mass of NH_3_^−^-N (mg) in 1 kg of soil after 12 h. CAT activity was assessed using the S-CAT activity test kit, where the volume of 0.1 mol/L potassium permanganate consumed after incubating 1 g of dry soil at 37 °C for 2 h was measured. POD activity was measured by the volumetric method. In test tubes containing the reaction mixture, enzyme solution was added, and the reaction was carried out for 1 h at 37 °C. The POD activity was calculated by measuring the absorbance change at 430 nm. PPO activity was determined using the iodine titration method. Enzyme solution was added to a buffer solution containing ortho-phenylenediamine, and the reaction was conducted for 3 h at 25 °C with 100 g of soil. The released iodine was titrated with sodium thiosulfate, and PPO activity was calculated. PP activity was determined using the phosphorus-benzene sodium colorimetric method. Soil samples were added to a buffer solution containing phosphorus-benzene sodium, and the mixture was incubated for 24 h at 37 °C. The reaction was terminated by adding aluminum sulfate solution, and after filtration, the filtrate was reacted with chlorinated dibromobenzene to form a blue color. The absorbance at 660 nm was measured, and phosphatase activity was calculated.

### Species diversity

According to the quadrat records, species diversity indexes, including the Shannon index, Pielou index, and Simpson's index, were calculated using the methods described in Ref.^115^ within R v4.2.3^116^. The Margalef richness index (*F*) was used to represent species richness, the Shannon–Wiener diversity index (*H'*) was used to represent species diversity, the Simpson dominance index (*D*) was used to represent species dominance, and the Pielou evenness index (*J*) was used to represent the uniformity of species distribution. The formulas for these indexes are as follows:

1 $$F=\frac{(S-1)}{\mathrm{ln }N}$$

2 $$H{\prime}=-\sum_{i-1}^{s}{P}_{i}{\text{ln}}{P}_{i}$$

3 $$D=1-\sum_{i}^{s}{P}_{i}^{2}$$

4 $$J=\frac{H{\prime}}{{\text{ln}}S}$$

In the formulas: *S* is the total number of species present within the quadrat; *N* represents the total number of individuals of all plants; *P*_*i*_ = *N*_*i*_*/N*, where *N*_*i*_ is the total number of individuals of the species *i*, and *P*_*i*_ is the relative importance value of species *i*.

### Community stability

Plant community stability was assessed using the inverse of the coefficient of variation (ICV)^117,118^. The formula as follows:

5 $${\text{ICV}}=\frac{\mu }{\sigma }$$

ICV is calculated as the ratio of the average density *μ* of each species in the quadrat to the standard deviation *σ* of their respective densities. A greater ICV value indicates heightened community stability and reduced variability in species density.

### Data analysis

The Shapiro–Wilk test was employed to assess whether the variables had undergone appropriate transformations to fulfill normality criteria and ensure the homogeneity of variances. A two-way analysis of variance (ANOVA) was employed to assess the variation in soil mixture properties resulting from the influence of restoration time (5a, 15a, 23a, and climax) and soil depth (0—10 cm, 10—20 cm, and 20—40 cm). Nonparametric statistical procedures were applied for variables with nonnormal residuals, the Kruskal–Wallis test was used, and the Dunn test in “PMCMRplus” packages^119^ was used for post-hoc comparison. Pearson’s correlation analysis was employed to examine the relationship between species diversity and soil mixture properties. Furthermore, the significant effects of soil properties and species diversity were analyzed using the Mantel test^120^ with package ‘ggcor’.

Partial Least Square Path Modeling (PLS-PM) was employed to confirm the correlations among soil enzyme activities, soil properties, and species diversity. The model underwent a process of filtration wherein non-significant manifest variables (with factor loadings less than 0.7) and certain non-significant paths were eliminated, with the objective of augmenting the model's goodness of fit and attaining an elevated level of confidence. For all of the reflection indicator loadings, > 0.7 was considered to be an appropriate reflection indicator for the corresponding latent variables^121^. The coefficient of determination (R^2^) represents the extent of variance in the dependent latent variable that is explained by its independent latent variables. A higher R^2^ value indicates a more robust model. In the context of PLS-PM, R^2^ signifies the percentage by which the constructs predict the dependent variable^122^. Several PLS-PM iterations were conducted to establish robust constructs. The goodness-of-fit (GOF) index was used to evaluate the model quality, which is calculated as the geometric mean of the average communality and average R^2^^121^. The PLS-PM analysis was conducted by using the “plspm” package in R 4.2.3.

### Supplementary Information

Supplementary Information.

## Data Availability

The datasets generated during and/or analyzed during the current study are available from the corresponding authors on reasonable request.
