# Supplementary material for: Effect of plant-soil system on the restoration of community stability after wildfire in the northeast margin of Qinghai-Tibet plateau
Source: Sci Rep. 2024 May 10;14:10706. doi: 10.1038/s41598-024-61621-2 (PMC11087542; doi:10.1038/s41598-024-61621-2)
Supplement: Supplementary file 1 — Supplementary Information. [file 41598_2024_61621_MOESM1_ESM.docx]

Supplementary Material

# 1 Supplementary Tables

**Table S1.** List of plants in the study area

| SPECIES | GENUS | FAMILY |
| --- | --- | --- |
| *Ligularia botryodes* | *Ligularia* | Asteraceae |
| *Ligularia sagitta* | *Ligularia* | Asteraceae |
| *Ligularia przewalskii* | *Ligularia* | Asteraceae |
| *Anaphalis sinica* | *Anaphalis* | Asteraceae |
| *Anaphalis nepalensis* | *Anaphalis* | Asteraceae |
| *Aster alpinus* | *Aster* | Asteraceae |
| *Aster ageratoides* | *Aster* | Asteraceae |
| *Sinosenecio oldhamianus* | *Sinosenecio* | Asteraceae |
| *Saussurea oligantha* | *Saussurea* | Asteraceae |
| *Saussurea amara* | *Saussurea* | Asteraceae |
| *Saussurea acroura* | *Saussurea* | Asteraceae |
| *Saussurea japonica* | *Saussurea* | Asteraceae |
| *Cirsium arvense* var*. integrifolium* | *Cirsium* | Asteraceae |
| *Parasenecio forrestii* | *Parasenecio* | Asteraceae |
| *Parasenecio roborowskii* | *Parasenecio* | Asteraceae |
| *Senecio nemorensis* | *Senecio* | Asteraceae |
| *Saussurea macrota* | *Saussurea* | Asteraceae |
| *Carpesium cernuum* | *Carpesium* | Asteraceae |
| *Lactuca indica* | *Lactuca* | Asteraceae |
| *Artemisia leucophylla* | *Artemisia* | Asteraceae |
| *Artemisia caruifolia* | *Artemisia* | Asteraceae |
| *Ajania potaninii* | *Ajania* | Asteraceae |
| *Artemisia argyi* | *Artemisia* | Asteraceae |
| *Picris hieracioides* | *Picris* | Asteraceae |
| *Cystopteris montana* | *Cystopteris* | Cystopteridaceae |
| *Athyrium sinense* | *Athyrium* | Athyriaceae |
| *Carex crebra* | *Carex* | Cyperaceae |
| *Thalictrum przewalskii* | *Thalictrum* | Ranunculaceae |
| *Clematis florida* | *Clematis* | Ranunculaceae |
| *Ranunculus tanguticus* | *Ranunculus* | Ranunculaceae |
| *Semiaquilegia adoxoides* | *Semiaquilegia* | Ranunculaceae |
| *Clematis macropetala* | *Clematis* | Ranunculaceae |
| *Clematis montana* | *Clematis* | Ranunculaceae |
| *Thalictrum callianthum* | *Thalictrum* | Ranunculaceae |
| *Caltha palustris* | *Caltha* | Ranunculaceae |
| *Actaea cimicifuga* | *Actaea* | Ranunculaceae |
| *Clematis tangutica* | *Clematis* | Ranunculaceae |
| *Thalictrum minus* | *Thalictrum* | Ranunculaceae |
| *Anemone rivularis* | *Anemone* | Ranunculaceae |
| *Thalictrum aquilegiifolium* var*. sibiricum* | *Thalictrum* | Ranunculaceae |
| *Aconitum tanguticum* | *Aconitum* | Ranunculaceae |
| *Aconitum henryi* | *Aconitum* | Ranunculaceae |
| *Actaea simplex* | *Actaea* | Ranunculaceae |
| *Anemone exigua* | *Anemone* | Ranunculaceae |
| *Rosa omeiensis* | *Rosa* | Rosaceae |
| *Rosa willmottiae* | *Rosa* | Rosaceae |
| *Rosa sweginzowii* | *Rosa* | Rosaceae |
| *Fragaria orientalis* | *Fragaria* | Rosaceae |
| *Rosa tsinglingensis* | *Rosa* | Rosaceae |
| *Fragaria vesca* | *Fragaria* | Rosaceae |
| *Cotoneaster acutifolius* | *Cotoneaster* | Rosaceae |
| *Geum aleppicum* | *Geum* | Rosaceae |
| *Rubus amabilis* | *Rubus* | Rosaceae |
| *Potentilla chinensis* | *Potentilla* | Rosaceae |
| *Spiraea salicifolia* | *Spiraea* | Rosaceae |
| *Sorbaria sorbifolia* | *Sorbaria* | Rosaceae |
| *Sorbus koehneana* | *Sorbus* | Rosaceae |
| *Cotoneaster acuminatus* | *Cotoneaster* | Rosaceae |
| *Rubus pileatus* | *Rubus* | Rosaceae |
| *Gentiana macrophylla* | *Gentiana* | Gentianaceae |
| *Halenia vaniotii* | *Halenia* | Gentianaceae |
| *Epilobium pyrricholophum* | *Epilobium* | Onagraceae |
| *Circaea alpina* | *Circaea* | Onagraceae |
| *Amsonia tabernaemontana* | *Amsonia* | Apocynaceae |
| *Caragana sinica* | *Caragana* | Fabaceae |
| *Vicia hirsuta* | *Vicia* | Fabaceae |
| *Vicia unijuga* | *Vicia* | Fabaceae |
| *Polygonatum odoratum* | *Polygonatum* | Asparagaceae |
| *Maianthemum bifolium* | *Maianthemum* | Asparagaceae |
| *Plantago asiatica* | *Plantago* | Plantaginaceae |
| *Veronica polita* | *Veronica* | Plantaginaceae |
| *Plantago depressa* | *Plantago* | Plantaginaceae |
| *Veronica vandellioides* | *Veronica* | Plantaginaceae |
| *Pedicularis torta* | *Pedicularis* | Orobanchaceae |
| *Pedicularis sylvatica* | *Pedicularis* | Orobanchaceae |
| *Pedicularis spicata* | *Pedicularis* | Orobanchaceae |
| *Galium boreale* | *Galium* | Rubiaceae |
| *Galium kinuta* | *Galium* | Rubiaceae |
| *Galium linearifolium* | *Galium* | Rubiaceae |
| *Rubia cordifolia* | *Rubia* | Rubiaceae |
| *Galium spurium* | *Galium* | Rubiaceae |
| *Polemonium caeruleum* | *Polemonium* | Polemoniaceae |
| *Polemonium chinense* | *Polemonium* | Polemoniaceae |
| *Geranium wilfordii* | *Geranium* | Geraniaceae |
| *Geranium nepalense* | *Geranium* | Geraniaceae |
| *Sanicula chinensis* | *Sanicula* | Apiaceae |
| *Pternopetalum heterophyllum* | *Pternopetalum* | Apiaceae |
| *Centella asiatica* | *Centella* | Apiaceae |
| *Torilis scabra* | *Torilis* | Apiaceae |
| *Heracleum moellendorffii* | *Heracleum* | Apiaceae |
| *Hansenia forbesii* | *Hansenia* | Apiaceae |
| *Torilis japonica* | *Torilis* | Apiaceae |
| *Anthriscus sylvestris* | *Anthriscus* | Apiaceae |
| *Urtica cannabina* | *Urtica* | Urticaceae |
| *Urtica fissa* | *Urtica* | Urticaceae |
| *Cardamine macrophylla* | *Cardamine* | Brassicaceae |
| *Chrysosplenium qinlingense* | *Chrysosplenium* | Saxifragaceae |
| *Chrysosplenium pilosum* | *Chrysosplenium* | Saxifragaceae |
| *Tiarella polyphylla* | *Tiarella* | Saxifragaceae |
| *Chenopodium album* | *Chenopodium* | Amaranthaceae |
| *Oxybasis glauca* | *Oxybasis* | Amaranthaceae |
| *Mentha haplocalyx* | *Mentha* | Lamiaceae |
| *Leonurus japonicus* | *Leonurus* | Lamiaceae |
| *Phlomoides umbrosa* | *Phlomoides* | Lamiaceae |
| *Salvia miltiorrhiza* | *Salvia* | Lamiaceae |
| *Primula polyneura* | *Primula* | Primulaceae |
| *Androsace umbellata* | *Androsace* | Primulaceae |
| *Fagopyrum dibotrys* | *Fagopyrum* | Polygonaceae |
| [*Persicaria lapathifolia*](http://www.iplant.cn/info/Persicaria%20lapathifolia) | *Persicaria* | Polygonaceae |
| *Koenigia pilosa* var*. hubertii* | *Koenigia* | Polygonaceae |
| *Bistorta vivipara* | *Bistorta* | Polygonaceae |
| *Persicaria nepalensis* | *Persicaria* | Polygonaceae |
| *Lonicera microphylla* | *Lonicera* | Caprifoliaceae |
| *Lonicera maackii* | *Lonicera* | Caprifoliaceae |
| *Lonicera trichosantha* | *Lonicera* | Caprifoliaceae |
| *Triosteum pinnatifidum* | *Triosteum* | Caprifoliaceae |
| *Lonicera caerulea* | *Lonicera* | Caprifoliaceae |
| *Lonicera ferdinandi* | *Lonicera* | Caprifoliaceae |
| *Lonicera nervosa* | *Lonicera* | Caprifoliaceae |
| *Lonicera japonica* | *Lonicera* | Caprifoliaceae |
| *Zabelia biflora* | *Zabelia* | Caprifoliaceae |
| *Lonicera tangutica* | *Lonicera* | Caprifoliaceae |
| *Lithospermum erythrorhizon* | *Lithospermum* | Boraginaceae |
| *Goodyera schlechtendaliana* | *Goodyera* | Orchidaceae |
| *Pseudocodon convolvulaceus* subsp*. forrestii* | *Pseudocodon* | Campanulaceae |
| *Allium victorialis* | *Allium* | Amaryllidaceae |
| *Asarum himalaicum* | *Asarum* | Aristolochiaceae |
| *Sambucus javanica* | *Sambucus* | Adoxaceae |
| *Viburnum dilatatum* | *Viburnum* | Adoxaceae |
| *Adoxa moschatellina* | *Adoxa* | Adoxaceae |
| *Sambucus williamsii* | *Sambucus* | Adoxaceae |
| *Sambucus adnata* | *Sambucus* | Adoxaceae |
| *Oxalis corniculata* | *Oxalis* | Oxalidaceae |
| *Oxalis pes caprae* | *Oxalis* | Oxalidaceae |
| *Euphorbia pekinensis* | *Euphorbia* | Euphorbiaceae |
| *Stellaria nemorum* | *Stellaria* | Caryophyllaceae |
| *Stellaria media* | *Stellaria* | Caryophyllaceae |
| *Poa nemoralis* | *Poa* | Poaceae |
| *Fargesia spathacea* | *Fargesia* | Poaceae |
| *Agrostis hugoniana* | *Agrostis* | Poaceae |
| *Juncus effusus* | *Juncus* | Juncaceae |
| *Hylotelephium angustum* | *Hylotelephium* | Crassulaceae |
| *Eleutherococcus senticosus* | *Eleutherococcus* | Araliaceae |
| *Aralia elata* | *Aralia* | Araliaceae |
| *Salix chaenomeloides* | *Salix* | Salicaceae |
| *Salix caprea* | *Salix* | Salicaceae |
| *Salix heishuiensis* | *Salix* | Salicaceae |
| *Salix cupularis* | *Salix* | Salicaceae |
| *Populus davidiana* | *Populus* | Salicaceae |
| *Philadelphus incanus* | *Philadelphus* | Hydrangeaceae |
| *Hydrangea bretschneideri* | *Hydrangea* | Hydrangeaceae |
| *Hydrangea paniculata* | *Hydrangea* | Hydrangeaceae |
| *Ribes burejense* | *Ribes* | Grossulariaceae |
| *Ribes stenocarpum* | *Ribes* | Grossulariaceae |
| *Ribes meyeri* | *Ribes* | Grossulariaceae |
| *Ribes janczewskii* | *Ribes* | Grossulariaceae |
| *Ribes pulchellum* | *Ribes* | Grossulariaceae |
| *Ribes alpestre* var*. giganteum* | *Ribes* | Grossulariaceae |
| *Smilax glaucochina* | *Smilax* | Smilacaceae |
| *Smilax menispermoidea* | *Smilax* | Smilacaceae |
| *Smilax discotis* | *Smilax* | Smilacaceae |
| *Clematoclethra scandens* | *Clematoclethra* | Actinidiaceae |
| *Berberis kansuensis* | *Berberis* | Berberidaceae |
| *Euonymus alatus* | *Euonymus* | Celastraceae |
| *Betula albosinensis* | *Betula* | Betulaceae |
| *Betula platyphylla* | *Betula* | Betulaceae |
| *Quercus mongolica* | *Quercus* | Fagaceae |
| *Quercus aliena* var*. acutiserrata* | *Quercus* | Fagaceae |
| *Picea crassifolia* | *Picea* | Pinaceae |
| *Picea asperata* | *Picea* | Pinaceae |
| *Picea purpurea* | *Picea* | Pinaceae |
| *Abies fargesii* var*. faxoniana* | *Abies* | Pinaceae |
| *Juniperus tibetica* | *Juniperus* | Cupressaceae |
| *Platycladus orientalis* | *Platycladus* | Cupressaceae |

**Table S2** Mantel test results of soil chemical properties and plant diversity 5 years after fire

| spec | env | r | p.value | df_r | df_p |
| --- | --- | --- | --- | --- | --- |
| Stability | TN | -0.253 | 0.839 | < 0.25 | ≥ 0.05 |
| Stability | TP | -0.374 | 0.878 | < 0.25 | ≥ 0.05 |
| Stability | TK | -0.355 | 0.883 | < 0.25 | ≥ 0.05 |
| Stability | AP | 0.048 | 0.381 | < 0.25 | ≥ 0.05 |
| Stability | AK | 0.318 | 0.139 | 0.25 - 0.5 | ≥ 0.05 |
| Stability | HN_4_^+^-N | -0.026 | 0.492 | < 0.25 | ≥ 0.05 |
| Stability | NO_3_^-^-N | 0.009 | 0.428 | < 0.25 | ≥ 0.05 |
| Stability | PH | -0.052 | 0.578 | < 0.25 | ≥ 0.05 |
| Stability | SOC | 0.005 | 0.431 | < 0.25 | ≥ 0.05 |
| Stability | C/N | 0.427 | 0.061 | 0.25 - 0.5 | ≥ 0.05 |
| Stability | C/P | 0.369 | 0.117 | 0.25 - 0.5 | ≥ 0.05 |
| Stability | N/P | -0.284 | 0.808 | < 0.25 | ≥ 0.05 |
| Stability | PP | -0.129 | 0.567 | < 0.25 | ≥ 0.05 |
| Stability | PPO | -0.259 | 0.911 | < 0.25 | ≥ 0.05 |
| Stability | URE | -0.035 | 0.444 | < 0.25 | ≥ 0.05 |
| Stability | POD | 0.080 | 0.317 | < 0.25 | ≥ 0.05 |
| Stability | CAT | 0.397 | 0.144 | 0.25 - 0.5 | ≥ 0.05 |
| Species diversity | TN | 0.048 | 0.403 | < 0.25 | ≥ 0.05 |
| Species diversity | TP | -0.341 | 0.908 | < 0.25 | ≥ 0.05 |
| Species diversity | TK | 0.421 | 0.063 | 0.25 - 0.5 | ≥ 0.05 |
| Species diversity | AP | 0.482 | 0.022 | 0.25 - 0.5 | 0.01 - 0.05 |
| Species diversity | AK | 0.560 | 0.013 | ≥ 0.5 | 0.01 - 0.05 |
| Species diversity | HN_4_^+^-N | -0.414 | 0.958 | < 0.25 | ≥ 0.05 |
| Species diversity | NO_3_^-^-N | -0.031 | 0.543 | < 0.25 | ≥ 0.05 |
| Species diversity | PH | 0.185 | 0.313 | < 0.25 | ≥ 0.05 |
| Species diversity | SOC | 0.107 | 0.342 | < 0.25 | ≥ 0.05 |
| Species diversity | C/N | 0.435 | 0.039 | 0.25 - 0.5 | 0.01 - 0.05 |
| Species diversity | C/P | 0.205 | 0.283 | < 0.25 | ≥ 0.05 |
| Species diversity | N/P | -0.103 | 0.589 | < 0.25 | ≥ 0.05 |
| Species diversity | PP | 0.255 | 0.178 | 0.25 - 0.5 | ≥ 0.05 |
| Species diversity | PPO | -0.116 | 0.653 | < 0.25 | ≥ 0.05 |
| Species diversity | URE | -0.188 | 0.732 | < 0.25 | ≥ 0.05 |
| Species diversity | POD | -0.193 | 0.732 | < 0.25 | ≥ 0.05 |
| Species diversity | CAT | 0.056 | 0.399 | < 0.25 | ≥ 0.05 |

**Table S3** Mantel test results of soil chemical properties and plant diversity 15 years after fire

| spec | env | r | p.value | df_r | df_p |
| --- | --- | --- | --- | --- | --- |
| Stability | TN | 0.469 | 0.053 | 0.25 - 0.5 | ≥ 0.05 |
| Stability | TP | 0.424 | 0.057 | 0.25 - 0.5 | ≥ 0.05 |
| Stability | TK | 0.131 | 0.267 | < 0.25 | ≥ 0.05 |
| Stability | AP | 0.305 | 0.131 | 0.25 - 0.5 | ≥ 0.05 |
| Stability | AK | 0.465 | 0.032 | 0.25 - 0.5 | 0.01 - 0.05 |
| Stability | HN_4_^+^-N | 0.051 | 0.458 | < 0.25 | ≥ 0.05 |
| Stability | NO_3_^-^-N | 0.319 | 0.126 | 0.25 - 0.5 | ≥ 0.05 |
| Stability | PH | -0.373 | 0.929 | < 0.25 | ≥ 0.05 |
| Stability | SOC | 0.349 | 0.147 | 0.25 - 0.5 | ≥ 0.05 |
| Stability | C/N | -0.261 | 0.775 | < 0.25 | ≥ 0.05 |
| Stability | C/P | 0.217 | 0.210 | < 0.25 | ≥ 0.05 |
| Stability | N/P | -0.104 | 0.643 | < 0.25 | ≥ 0.05 |
| Stability | PP | -0.280 | 0.824 | < 0.25 | ≥ 0.05 |
| Stability | PPO | 0.135 | 0.321 | < 0.25 | ≥ 0.05 |
| Stability | URE | 0.086 | 0.457 | < 0.25 | ≥ 0.05 |
| Stability | POD | 0.217 | 0.215 | < 0.25 | ≥ 0.05 |
| Stability | CAT | -0.071 | 0.593 | < 0.25 | ≥ 0.05 |
| Species diversity | TN | 0.102 | 0.386 | < 0.25 | ≥ 0.05 |
| Species diversity | TP | -0.122 | 0.722 | < 0.25 | ≥ 0.05 |
| Species diversity | TK | 0.365 | 0.115 | 0.25 - 0.5 | ≥ 0.05 |
| Species diversity | AP | -0.142 | 0.724 | < 0.25 | ≥ 0.05 |
| Species diversity | AK | -0.049 | 0.579 | < 0.25 | ≥ 0.05 |
| Species diversity | HN_4_^+^-N | 0.213 | 0.246 | < 0.25 | ≥ 0.05 |
| Species diversity | NO_3_^-^-N | 0.172 | 0.269 | < 0.25 | ≥ 0.05 |
| Species diversity | PH | -0.040 | 0.550 | < 0.25 | ≥ 0.05 |
| Species diversity | SOC | -0.201 | 0.783 | < 0.25 | ≥ 0.05 |
| Species diversity | C/N | 0.164 | 0.308 | < 0.25 | ≥ 0.05 |
| Species diversity | C/P | -0.243 | 0.799 | < 0.25 | ≥ 0.05 |
| Species diversity | N/P | 0.095 | 0.343 | < 0.25 | ≥ 0.05 |
| Species diversity | PP | 0.058 | 0.432 | < 0.25 | ≥ 0.05 |
| Species diversity | PPO | 0.351 | 0.132 | 0.25 - 0.5 | ≥ 0.05 |
| Species diversity | URE | 0.319 | 0.161 | 0.25 - 0.5 | ≥ 0.05 |
| Species diversity | POD | 0.329 | 0.150 | 0.25 - 0.5 | ≥ 0.05 |
| Species diversity | CAT | 0.065 | 0.418 | < 0.25 | ≥ 0.05 |

**Table S4** Mantel test results of soil chemical properties and plant diversity 23 years after fire

| spec | env | r | p.value | df_r | df_p |
| --- | --- | --- | --- | --- | --- |
| Stability | TN | 0.132 | 0.383 | < 0.25 | ≥ 0.05 |
| Stability | TP | 0.008 | 0.558 | < 0.25 | ≥ 0.05 |
| Stability | TK | 0.078 | 0.308 | < 0.25 | ≥ 0.05 |
| Stability | AP | 0.037 | 0.458 | < 0.25 | ≥ 0.05 |
| Stability | AK | 0.117 | 0.550 | < 0.25 | ≥ 0.05 |
| Stability | HN_4_^+^-N | -0.276 | 0.742 | < 0.25 | ≥ 0.05 |
| Stability | NO_3_^-^-N | 0.415 | 0.267 | 0.25 - 0.5 | ≥ 0.05 |
| Stability | PH | -0.449 | 0.967 | < 0.25 | ≥ 0.05 |
| Stability | SOC | 0.150 | 0.292 | < 0.25 | ≥ 0.05 |
| Stability | C/N | -0.381 | 0.892 | < 0.25 | ≥ 0.05 |
| Stability | C/P | 0.494 | 0.167 | 0.25 - 0.5 | ≥ 0.05 |
| Stability | N/P | -0.022 | 0.500 | < 0.25 | ≥ 0.05 |
| Stability | PP | -0.164 | 0.658 | < 0.25 | ≥ 0.05 |
| Stability | PPO | 0.455 | 0.008 | 0.25 - 0.5 | < 0.01 |
| Stability | URE | -0.132 | 0.675 | < 0.25 | ≥ 0.05 |
| Stability | POD | -0.180 | 0.533 | < 0.25 | ≥ 0.05 |
| Stability | CAT | -0.170 | 0.725 | < 0.25 | ≥ 0.05 |
| Species diversity | TN | 0.071 | 0.442 | < 0.25 | ≥ 0.05 |
| Species diversity | TP | -0.074 | 0.592 | < 0.25 | ≥ 0.05 |
| Species diversity | TK | 0.123 | 0.342 | < 0.25 | ≥ 0.05 |
| Species diversity | AP | 0.067 | 0.392 | < 0.25 | ≥ 0.05 |
| Species diversity | AK | 0.079 | 0.483 | < 0.25 | ≥ 0.05 |
| Species diversity | HN_4_^+^-N | -0.293 | 0.808 | < 0.25 | ≥ 0.05 |
| Species diversity | NO_3_^-^-N | 0.276 | 0.300 | 0.25 - 0.5 | ≥ 0.05 |
| Species diversity | PH | -0.459 | 0.917 | < 0.25 | ≥ 0.05 |
| Species diversity | SOC | -0.121 | 0.608 | < 0.25 | ≥ 0.05 |
| Species diversity | C/N | -0.588 | 0.958 | < 0.25 | ≥ 0.05 |
| Species diversity | C/P | 0.388 | 0.250 | 0.25 - 0.5 | ≥ 0.05 |
| Species diversity | N/P | -0.284 | 0.742 | < 0.25 | ≥ 0.05 |
| Species diversity | PP | -0.047 | 0.500 | < 0.25 | ≥ 0.05 |
| Species diversity | PPO | 0.556 | 0.008 | ≥ 0.5 | < 0.01 |
| Species diversity | URE | -0.178 | 0.667 | < 0.25 | ≥ 0.05 |
| Species diversity | POD | -0.182 | 0.683 | < 0.25 | ≥ 0.05 |
| Species diversity | CAT | -0.114 | 0.558 | < 0.25 | ≥ 0.05 |

**Table S5** Mantel test results of soil chemical properties and plant diversity in climax communities

| spec | env | r | p.value | df_r | df_p |
| --- | --- | --- | --- | --- | --- |
| Stability | TN | -0.050 | 0.520 | < 0.25 | ≥ 0.05 |
| Stability | TP | -0.011 | 0.467 | < 0.25 | ≥ 0.05 |
| Stability | TK | -0.158 | 0.863 | < 0.25 | ≥ 0.05 |
| Stability | AP | -0.058 | 0.572 | < 0.25 | ≥ 0.05 |
| Stability | AK | 0.112 | 0.201 | < 0.25 | ≥ 0.05 |
| Stability | HN_4_^+^-N | 0.026 | 0.307 | < 0.25 | ≥ 0.05 |
| Stability | NO_3_^-^-N | -0.207 | 0.918 | < 0.25 | ≥ 0.05 |
| Stability | PH | -0.108 | 0.711 | < 0.25 | ≥ 0.05 |
| Stability | SOC | 0.128 | 0.192 | < 0.25 | ≥ 0.05 |
| Stability | C_N | -0.051 | 0.499 | < 0.25 | ≥ 0.05 |
| Stability | C/P | 0.027 | 0.339 | < 0.25 | ≥ 0.05 |
| Stability | N/P | -0.056 | 0.583 | < 0.25 | ≥ 0.05 |
| Stability | PP | -0.021 | 0.361 | < 0.25 | ≥ 0.05 |
| Stability | PPO | -0.025 | 0.480 | < 0.25 | ≥ 0.05 |
| Stability | URE | -0.092 | 0.642 | < 0.25 | ≥ 0.05 |
| Stability | POD | -0.142 | 0.785 | < 0.25 | ≥ 0.05 |
| Stability | CAT | -0.103 | 0.688 | < 0.25 | ≥ 0.05 |
| Species diversity | TN | 0.064 | 0.389 | < 0.25 | ≥ 0.05 |
| Species diversity | TP | 0.057 | 0.321 | < 0.25 | ≥ 0.05 |
| Species diversity | TK | 0.002 | 0.431 | < 0.25 | ≥ 0.05 |
| Species diversity | AP | 0.190 | 0.159 | < 0.25 | ≥ 0.05 |
| Species diversity | AK | -0.128 | 0.646 | < 0.25 | ≥ 0.05 |
| Species diversity | HN_4_^+^-N | -0.173 | 0.820 | < 0.25 | ≥ 0.05 |
| Species diversity | NO_3_^-^-N | -0.079 | 0.607 | < 0.25 | ≥ 0.05 |
| Species diversity | PH | 0.479 | 0.031 | 0.25 - 0.5 | 0.01 - 0.05 |
| Species diversity | SOC | 0.309 | 0.094 | 0.25 - 0.5 | ≥ 0.05 |
| Species diversity | C/N | -0.204 | 0.802 | < 0.25 | ≥ 0.05 |
| Species diversity | C/P | 0.460 | 0.037 | 0.25 - 0.5 | 0.01 - 0.05 |
| Species diversity | N/P | 0.349 | 0.096 | 0.25 - 0.5 | ≥ 0.05 |
| Species diversity | PP | -0.122 | 0.650 | < 0.25 | ≥ 0.05 |
| Species diversity | PPO | 0.710 | 0.006 | ≥ 0.5 | < 0.01 |
| Species diversity | URE | 0.023 | 0.363 | < 0.25 | ≥ 0.05 |
| Species diversity | POD | -0.098 | 0.700 | < 0.25 | ≥ 0.05 |
| Species diversity | CAT | 0.386 | 0.057 | 0.25 - 0.5 | ≥ 0.05 |

**Table S6** Basic situation of communities and the sampling sites at different succession stages

| Succession stages | Forest station | Year of forest fire | Fire intensity | Management and protection measures | Slope (°) | Altitude(m) | Soil type | Depth of soil layer (cm) | Composition of community |
| --- | --- | --- | --- | --- | --- | --- | --- | --- | --- |
| 5a | Dala | 2016 | High fire intensity | Closure | 30~44 | 2581~2870 | Brown soil | 40~200 | 90 species 66 genus in 34 families |
| 15a | Yiwa | 2005 | High fire intensity | Closure | 22~52 | 2981~ 3390 | Gray brown soil | 49~107 | 77 species 59 genus in 31 families |
| 23a | Nagai | 1997 | High fire intensity | Closure，litters cleared out in 2011 | 27~ 38 | 2680~ 3100 | Gray brown soil | 52~210 | 70 species 48 genus in 31 families |
| Climax community | Yiwa, Dala and Nagai | No | No | Closure | 21~ 54 | 2575~ 3410 | Brown soil，gray brown soil | 36~308 | 105 species 72 genus in 38 families |

**Table S7** Direct, indirect, and total effects between modeled variables within different factors in PLS-PM in 5a, 15a, 23a, and climax community.

| Community | Relationships | Direct | Indirect | Total |
| --- | --- | --- | --- | --- |
| 5a | Soil characteristics - Enzyme activity | -0.938 | 0.000 | -0.938 |
|  | Soil characteristics - Species diversity | -1.672 | 1.621 | -0.051 |
|  | Soil characteristics - Community stability | 1.641 | -0.903 | 0.738 |
|  | Enzyme activity- Species diversity | -1.728 | 0.000 | -1.728 |
|  | Enzyme activity- Community stability | 0.919 | -1.372 | -0.453 |
|  | Species diversity - Community stability | 0.794 | 0.000 | 0.794 |
| 15a | Soil characteristics - Enzyme activity | -0.865 | 0.000 | -0.865 |
|  | Soil characteristics - Species diversity | -0.811 | 0.969 | 0.159 |
|  | Soil characteristics - Community stability | 0.471 | -0.339 | 0.131 |
|  | Enzyme activity- Species diversity | -1.121 | 0.000 | -1.121 |
|  | Enzyme activity- Community stability | 0.587 | -1.189 | -0.602 |
|  | Species diversity - Community stability | 1.060 | 0.000 | 1.060 |
| 23a | Soil characteristics - Enzyme activity | -0.731 | 0.000 | -0.731 |
|  | Soil characteristics - Species diversity | 0.140 | 0.587 | 0.726 |
|  | Soil characteristics - Community stability | -0.595 | 0.968 | 0.373 |
|  | Enzyme activity- Species diversity | -0.802 | 0.000 | -0.802 |
|  | Enzyme activity- Community stability | 0.047 | -1.107 | -1.060 |
|  | Species diversity - Community stability | 1.380 | 0.000 | 1.380 |
| Climax | Soil characteristics - Enzyme activity | 0.894 | 0.000 | 0.894 |
|  | Soil characteristics - Species diversity | -0.167 | -0.498 | -0.665 |
|  | Soil characteristics - Community stability | 0.698 | -0.653 | 0.045 |
|  | Enzyme activity- Species diversity | -0.558 | 0.000 | -0.558 |
|  | Enzyme activity- Community stability | -0.121 | -0.457 | -0.578 |
|  | Species diversity - Community stability | 0.819 | 0.000 | 0.819 |

# 2 Supplementary figures


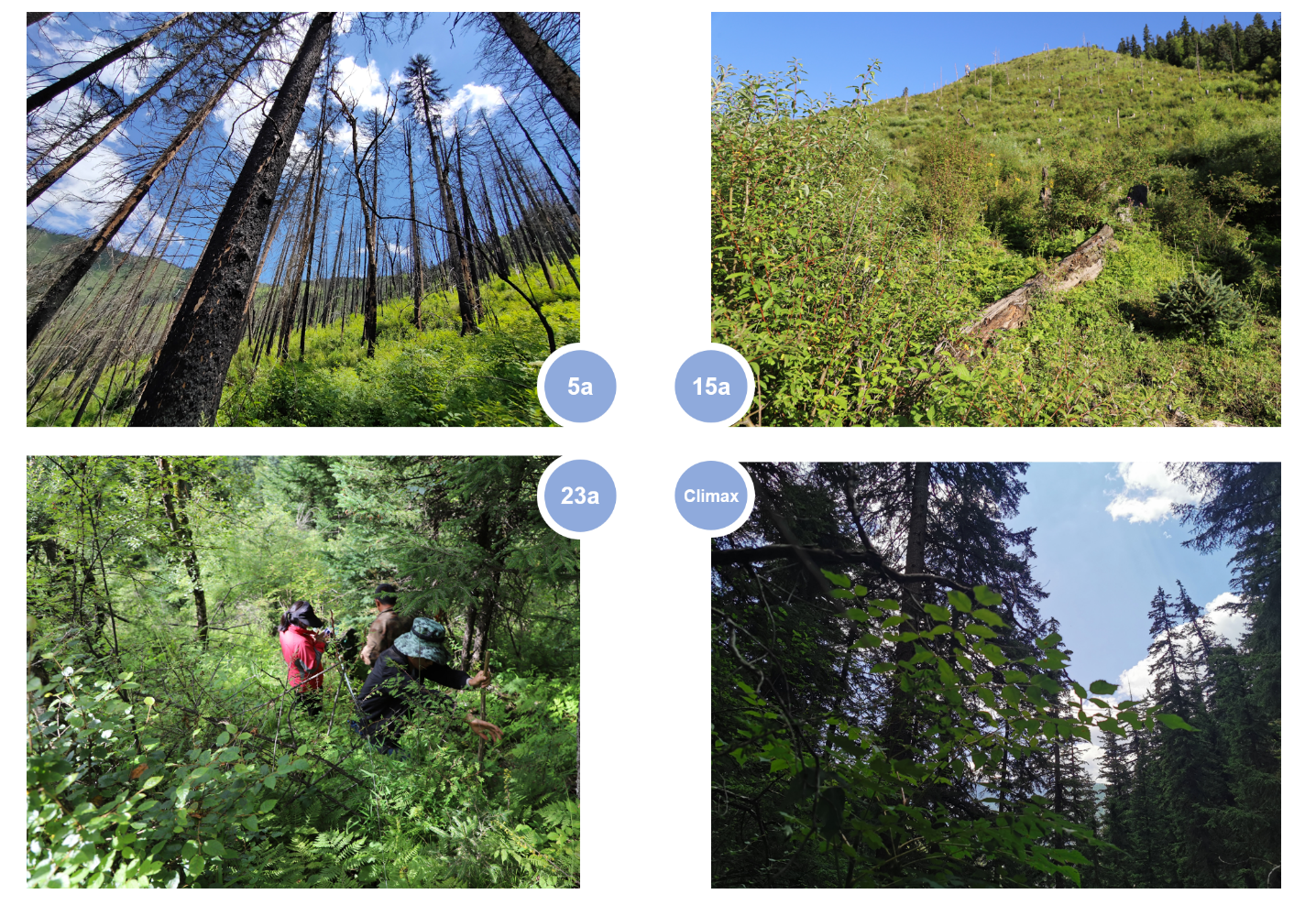


**Figure S1** Ecosystem photos of post-fire 5a, 13a, 23a, and climax communities.
